# 

**Published:** 1895-10-12

**Authors:** 


**CADBURY'S COCOA**

"Of absolute purity and freedom from alkali." —*Braithwaite.*

**CADBURY'S COCOA**

"The Typical Cocoa of English Manufacture. Absolutely Pure."—*The Analyst.*

### THE HOSPITAL

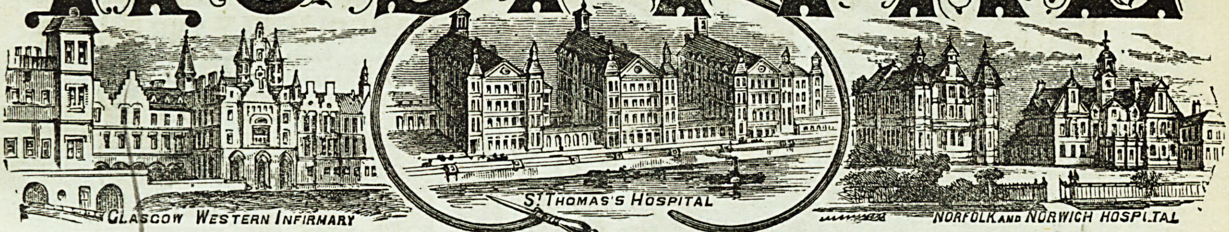

No. 472. Vol. XIX.

SATURDAY, OCT. 12TH, 1895.

**WITH EXTRA NURSING SUPPLEMENT.**

[Registered at the General Post Office as a Newspaper for transmission in the United Kingdom and Abroad.]

**PRICE TWOPENCE.**

Per Annum, 10s. 6d., post free.

Monthly Parts, 6d.; post free, 8d.

Ditto per Annum, 8s. 6d., post free.

